# Emergence of a Novel Reassortant Strain of Bluetongue Serotype 6 in Israel, 2017: Clinical Manifestations of the Disease and Molecular Characterization

**DOI:** 10.3390/v11070633

**Published:** 2019-07-10

**Authors:** Natalia Golender, Avi Eldar, Marcelo Ehrlich, Yevgeny Khinich, Gabriel Kenigswald, Joseph Seffi Varsano, Shachar Ertracht, Itzik Abramovitz, Itay Assis, Ily Shlamovitz, Eitan Tiomkin, Erez Yonay, Benny Sharir, Velizar Y. Bumbarov

**Affiliations:** 1 Kimron Veterinary Institute, Division of Virology, Beit Dagan 50250, Israel; 2 School of Molecular Cell Biology Biotechnology, Tel Aviv University, Tel Aviv 69978, Israel; 3 Hachaklait veterinary services, Caesarea 3088900, Israel; 4 Indi-Vet Ltd., Ashdod 7764933, Israel; 5 Dr. Amos Bareli LTD, Kiryat Tiv’on 3605141, Israel; 6 Lamikne veterinary services, Kiryat Tiv’on 3603247, Israel

**Keywords:** bluetongue virus, orbivirus, *Reoviridae*, sequencing, phylogenetic analysis, diagnostics, spread, descriptive epidemiology

## Abstract

Reassortment contributes to the evolution of RNA viruses with segmented genomes, including Bluetongue virus (BTV). Recently, co-circulation of natural and vaccine BTV variants in Europe, and their ensuing reassortment, were proposed to promote appearance of novel European BTV strains, with potential implications for pathogenicity, spread and vaccination policies. Similarly, the geographical features of the Mediterranean basin, which spans over portions of three continents, may facilitate the appearance of clinically relevant reassortants via co-circulation of BTV strains of African, Asian and European origins. In August–October 2017, BTV serotype 6 (BTV-6) was identified in young animals exhibiting classical clinical signs of Bluetongue (BT) at Israeli sheep and cattle farms. Sequencing and pairwise analysis of this Israeli BTV-6 isolate revealed the closest sequence homology of its serotype-defining Segment 2 was with that of South African reference BTV-6 strain 5011 (93.88% identity). In contrast, the other viral segments showed highest homology (97.0%–99.47% identity) with BTV-3, -4 and -9 of Mediterranean and African origins. Specifically, four viral segments were nearly identical (99.13%–99.47%), with Tunisian and Italian BTV-3 strains (TUN2016 and SAD2018, correspondingly). Together, our data suggest that Mediterranean co-circulation and reassortment of BTV-3 and BTV-6 drove the emergence of a novel and virulent BTV-6 strain

## 1. Introduction

Bluetongue (BT) is an arthropode-born disease of domestic and wild ruminants caused by the Bluetongue virus (BTV) [1,2]. The agent and its associated diseases present wide global distribution [2]. Indeed, BT is one of the major infectious diseases of ruminants and is listed as a notifiable disease by the World Organization for Animal Health (OIE). Transmission between mammalian hosts is mainly carried out by competent *Culicoides* species [3,4,5,6]. In addition to *Culicoides*-mediated transmission, a limited repertoire of BTV serotypes has recently been characterized as being able to undergo transplacental transmission, oral-based transmission or aerosol-contact based transmission [7]. Clinical signs of BT are generally most severe in naïve sheep and white-tailed deer, where death is not an uncommon outcome [1,2]. Of note, testicular degeneration and azoospermia were also recently identified as clinical features of BTV infection of rams [8], extending in this manner the range of putative damage which can be inflicted by BTV outbreaks. In contrast, BTV infection in cattle is usually asymptomatic, although it appears that some strains are more pathogenic and are able to induce a clinical disease. These cases are frequently characterized by a sharp reduction in milk production, depression, lethargy, pyrexia, lameness or stiff gait, serous/purulent nasal discharge, excessive salivation, facial edema, cyanosis/petechiae/erosions of the tongue and oral mucosa, and dyspnea [1,2,9,10,11].

The genome of BTV (family Reoviridae, genus Orbivirus) comprises ten segments (Seg-1 to Seg-10) of double-stranded linear RNA and codes for seven virus-structural (VP1–VP7) and four non-structural (NS1, NS2, NS3/NS3a and NS4) proteins, while the existence of a fifth non-structural protein has also been suggested [12]. Based on sequence analysis of outer protein VP2 (the most variable gene), twenty-seven distinct BTV serotypes have been recognized [7] and several others have been suggested [13,14,15,16]. Phylogenetic analysis points out the relatedness of several of these serotypes, as in the case of BTV-6, which is closely related to BTV-14 and to BTV-21 [17], while resembling to a lesser degree BTV-3, BTV-13 and BTV-16.

BTV-6 is widely distributed and has been identified in several continents, including the Americas (South and North America), the Caribbean, Africa and Asia [18,19,20,21,22]. Notably, in 2008, a reassortant live vaccine strain of BTV-6 was observed to induce clinical symptoms in cows in the Netherlands [23]. These observed cases support the notion of the pathogenic potential of BTV-6 in cattle, as well as serving as a cautionary tale of the putative influence of anthropogenic factors in the spread of BTV [2]. Indeed, a similar event was observed in Israel, where isolation of BTV-6 from clinical samples between 1972 and 1989 was linked to iatrogenic dissemination of the live attenuated vaccine strain [20].

Genetic recombination of RNA viruses with segmented genomes through reassortment involves the packaging, into a single virion, of genomic segments of different ancestry [24]. In order for such genome mixing to occur, a given cell needs to be simultaneously infected with more than one virus. Notably, in spite of the very large number of different combinations that can be generated by random reassortment of multiple segments (e.g., the 10 genomic dsRNA segments of BTV), reassortment-related packaging of genome segments is frequently non-random, thus effectively limiting the repertoire of reassorted virions (reviewed in [24]). Concerning BTV, recent studies support the following notions: (i) All 10 segments can undergo reassortment, which occurs readily upon co-cultivation in vitro and is also evidenced by genetic analysis of circulating BT strains [25,26,27,28]. (ii) Central characteristics of BTV virulence and/or interactions with the host cell (e.g., molecular preference during cell attachment, replication kinetics, ability to replicate in interferon-competent cells, mode and extent of induction of cell death) can be modulated through reassortment; underscoring the importance of reassortment for BTV evolution [25,26,27]. (iii) Reassortment is non-random in terms of the packaging of viral segments. For example, six of 10 segments (Seg-1, Seg-3, Seg-4, Seg-5, Seg-8, Seg-9) exhibit a bias towards co-packaging, suggestive of evolutionary links (e.g., epistatic interactions stemming from biochemical or physical interactions of the gene products encoded in these segments) [28].

In 2017, a severe BTV-6 outbreak was recorded throughout Israel. At the end of August 2017, BTV-6 simultaneously appeared in the northern and southern areas of Israel (Galilee and Southern Shfela districts, respectively) and quickly spread throughout the Golan Heights and the central areas of the country. Importantly, this outbreak was characterized by classical clinical manifestations of the disease and death in sheep and cattle.

The present paper describes the clinical outcomes of BTV-6 infection in Israeli sheep and cattle herds, along with the genetic characterization and phylogenetic analysis of this recently identified BTV-6 Israeli strain. Our results demonstrate the introduction of a new and genetically distinct BTV-6 strain in 2017. The novel genetic features of this novel strain, and their correlation with observed clinical symptoms, suggest an ever increasing significance of BTV-6 as a pathogen for domestic ruminants.

## 2. Materials and Methods

2.1. qRT-PCR Tests for Viruses Other Than BTV

During 2017, a febrile disease was observed in domestic and wild ruminants. A total of 214 cattle samples (212 whole blood EDTA samples, one spleen sample from a dead cow, and one spleen sample from aborted cattle fetus) were tested for bovine ephemeral fever virus (BEFV) by RT-qPCR [29] (Table 1). In addition, 226 field cattle samples (217 whole blood EDTA samples from ill cattle, six spleen samples from dead cattle and three aborted cattle fetuses (two spleen, one brain and one placenta samples)) and 8 samples from wild animals (spleen samples from five Nubian ibexes, one giraffe and two mountain gazelles; and one spleen sample from an aborted fetus of the fallow deer) were tested for hemorrhagic disease virus (EHDV) by qRT-PCR (Table 1). Briefly, RNA (for EHDV tests) was extracted from whole blood samples and tissue using Invisorb Spin Virus RNA Mini Kit (STRATEC Molecular GmbH, Berlin, Germany). EHDV RNA presence was assessed with an Epizootic Hemorrhagic Disease Virus Real-Time PCR Kit (LSI VetMAX, Lissieu, France), as previously described [30].

### 2.2. Pan-BTV qRT-PCR

RNA was extracted as previously described for EHDV [30]. A total of 693 field samples were included in the present study. These originated from 402 cattle whole blood EDTA samples, 35 spleens and 7 aborted cattle fetuses (four spleen, one brain, one placenta and one mixed sample, which included brain, spleen, liver and lung), 173 whole blood sheep EDTA samples, 41 spleens from dead sheep and 10 aborted sheep fetuses (seven spleen, two lung, two brain, and two mixed samples, which included brain, spleen, liver and lung). Whole blood samples from wild animals were obtained from camels (14 samples), elephants (3 samples); spleen samples were attained from 4 mountain gazelles, 5 roe deer, 3 Persian fallow deer, 9 Nubian ibexes, 1 alpaca; 2 aborted Persian fallow deer fetuses, 1 adult giraffe and 1 aborted giraffe fetus (Table 2). Initial assessment of BTV was performed by using VetMAX™ BTV NS3 All Genotypes Kit (Applied Biosystems™, Thermo Fisher Scientific Inc., France), as described by the manufacturer (referred to hereafter as Pan-BTV qRT-PCR). 

### 2.3. BT Virus Isolation.

RT-qPCR BTV positive samples (total of 166, Table 2) were inoculated onto 9–11 day old embryonated chicken eggs (ECE) by intravenous delivery, as previously described [30,31], with minor modifications. Briefly, tissue samples were prepared by the same method, which was described above, and supernatant from organ samples was filtrated using 0.45 µm filter (Starstedt, Germany) and stored at 4 °C until use. For isolating BTV from whole blood samples, red blood cells (RBC) were washed 3 times with PBS. Then, 100 µL of washed RBC were resuspended in 900 µL of double distillated water to induce hemolysis. A total of 100 µL from prepared samples were inoculated into each ECE (5 eggs per sample) and eggs were observed for 9 days. Dead ECE (between days 2–9), were homogenized. Supernatant from ECE homogenates was used for RNA extraction and was tested by pan-BTV RT-qPCR; positive in pan-BTV RT-qPCR ECE samples were subsequently passaged 3 or 4 times on baby hamster kidney cells (BHK-21) monolayers (until appearance of a cytopathic effect (CPE)).

### 2.4. BTV Serotype Identification

RNA from ECE homogenates was extracted using Invisorb Spin Virus RNA Mini Kit (STRATEC Molecular GmbH, Berlin, Germany) according to manufacturer’s instructions. ECE homogenates which were found positive by Pan-BTV RT-qPCR were further assessed by RT-PCR for the typing of serotypes 2, 3, 4, 5, 8, 12, 15, 16, 24 and 28 (all with in house developed primer pairs), which are known to be present in Israel and neighboring countries, using One-Step RT-PCR kit (Qiagen, Hilden, Germany). Identification of BTV-6 isolates was performed with the following pair of primers (developed in house): 6VP2-124F 5′- TGTAACCCAAATTCCCACGAA-3′ and 6VP2-1030R 5′-CAGAGGCGGCTATCATA-3′. Amplified fragments were subsequently sequenced.

### 2.5. Sequencing and Phylogenetic Analysis

Following three passages on BHK-21cells, the BTV strain (ISR-2095/3/17) was sequenced by NGS at Hy Laboratories Ltd., Rehovot, Israel. Sequence gaps were filled following the performance of conventional RT-PCR, employing in house designed primer-pairs. cDNA fragments were purified using MEGAquick-spin™ Total Fragment DNA Purification Kit (iNtRON Biotechnology, Gyeonggi-do, South Korea) and standard Sanger sequencing was performed on ABI 3730xl DNA Analyzer (Hy Laboratories Ltd., Rehovot, Israel).

Nucleotide sequences were assembled and nucleotide (nt) and amino acid (aa) sequences were aligned and pairwise compared using Geneious (version 9.0.5; Biomatters, Auckland, New Zealand). Phylogenetic trees were constructed using Mega 7.1 [32].

The ten segment codding regions of Israeli BTV-6 ISR-2095/17 isolate were mostly sequenced (Seg-1-positions 1-3934/3944; Seg-2- 17-2892/2922; Seg-3- 21-2738/2772; Seg-4- 17-1967/1981; Seg-5-2-1688/1772; Seg-6-2-1634/1637; Seg-7- 20-1127/1156; Seg-8- 2-1080/1125; Seg-9-13-1035/1067; Seg-10- 1-800/822) and submitted to NCBI GenBank (MH383089-MH383098). Seg-2 of all other BTV-6 isolated in 2017 were sequenced partially (MH791296-MH791313). Several segments (Seg-1, -3, -5, -6, -7 and -10) of some other BTV-6 isolates were also partially sequenced (MH426705-MH426723).

## 3. Results

### 3.1. Clinical Signs in Sheep and Cattle Naturally Infected with BTV-6

**Figure 1:**
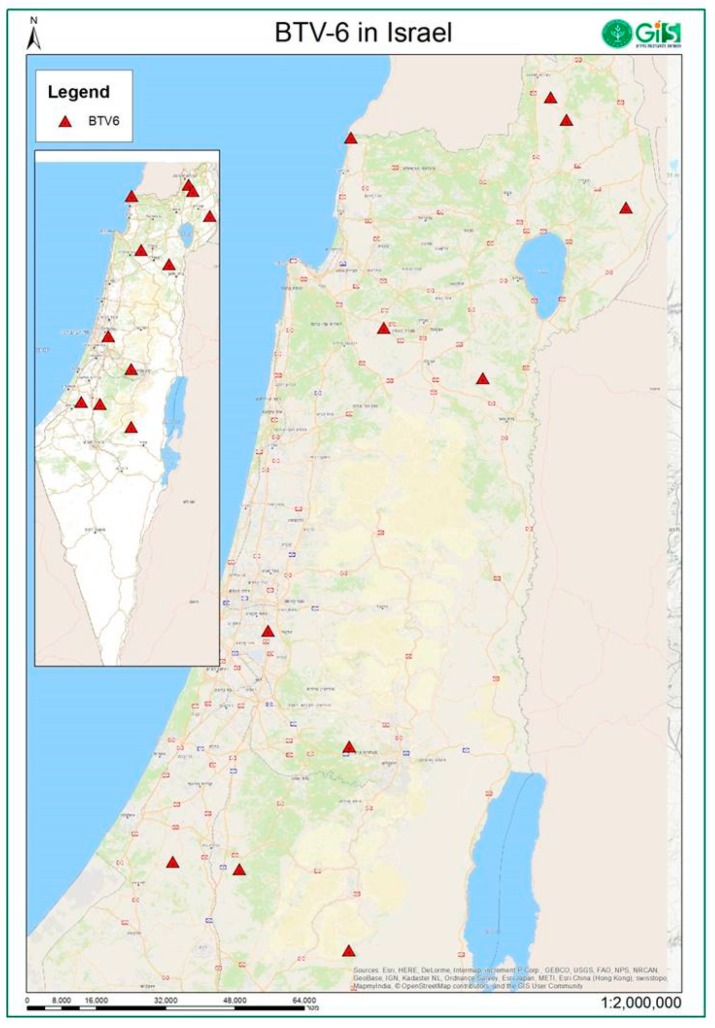
Location of BTV-6 affected sheep and cattle farms, Israel.

From the end of August until mid-October 2017, BTV-6 was identified in clinically affected sheep (*n* = 6) and cattle milking and fattening farms (*n* = 5) located in southern, central and northern parts of the county (Figure 1, Table 3 and Table 4). In Israeli sheep, BTV-6 was observed mostly in young animals ranging from 4 to 20 months of age, while clinical signs in adult animals were observed only in two farm (farms 5 and 10; Table 3 and Table 4). In young sheep, the major clinical sings included pyrexia, skin hyperemia, lameness, rejection of moving, stiffness in leg and back muscles, bloody nasal discharge, recumbency and breath abnormalities; in adult milking ewes clinical signs included milk reduction, heavy hyperemia of skin (udder, face), light perinasal and perioral edema. Morbidity in flocks ranged 17%–34% and was observed mostly in lambs. Mortality greatly varied between flocks (from 2% to 83% of identified cases) and appeared to depend on general flock welfare and on symptomatic treatment of ill animals. In cattle, clinical signs were observed in young animals only (ranging from three month to two years old). Inappetence, cachexia, dyspnea and cough were observed in calves for fattening. In addition, field post mortem examination revealed pneumonia in two dead calves. Udder edema, as well as symmetrical and bilateral edema throughout the length of the limbs, were observed in milking heifers, lasting for two weeks (Table 3).

### 3.2. BTV Detection and Isolation, 2017

To probe for presence of BTV, we carried out qRT-PCR analysis of field samples. Specifically, 166 out of 693 field samples were positive for BTV by qRT-PCR. Positive samples were found in diseased and dead cattle (99 of 444 samples), sheep (60 of 224 samples) and goat (6 of 33 samples). In addition, three weak positive results were observed from 53 tested samples from zoo and wild animals (one whole blood sample from elephant, one from mountain gazelle and one from Persian roe deer). The rarity of these findings, the low load of BTV RNA in these samples and our inability to isolate the virus or identify serotype, leads us to conclude that the role of wild animals and goats in BTV outbreaks 2017 was negligible. Moreover, we analyzed only a small number of samples from aborted fetuses (*n* = 20), preventing us from reaching substantive conclusions regarding the role of transplacental transmission in this recent BTV outbreak in Israel.

Overall, in 2017 forty BTV isolates belonging to serotypes 2, 3, 4, 6 and 15 were identified. BTV-2 was isolated from cattle in July 2017, BTV-4 was isolated from sheep and cattle between June and November 2017 in all parts of the country, BTV-3 was found in sheep from three different locations situated in the southern parts of Israel only during autumn months 2017, while BTV-15 was isolated from cattle in late-October–December 2017 from southern and central parts of Israel. In comparison to these relatively sparse cases, BTV-6 showed the highest proportion of virus isolation relative to all other serotypes during late-summer–fall 2017 (Table 2). To further and quantitatively characterize our findings, we opted to classify the samples according to their Ct value (Table 5). Specifically, we classified the positive samples as very strong positive (Ct < 25.0); strong positive (Ct 25.1–30.0) weak positive (Ct 30.1–35.0) and very weak positive (Ct > 35.0). According to the data, there were no “very strong positive” samples in the course of the initial four months of 2017 (winter months). These results are in accord with our inability to isolate BTV during this period of time, suggesting that the positive samples may stem from prolonged RNAmia of infections occurring in the previous year. Based on our data, we propose that the first cases of BTV infection of domestic animals in 2017 took place in May, where two samples were very strong positive (Table 5). Robust multiple-serotype outbreaks of BTV began in July 2017. Accordingly, August–October 2017 exhibited the highest proportion of positive results (BTV-infected animals compared to the total number of tested ill animals), reaching a peak of more than 50% in September 2017. The notion of a peak in the dynamics of the outbreak is also supported by the high proportion of successful virus isolation from very strong and strong positive samples (>50%). Following the October peak, the proportion of strong and very strong positive samples consequently decreased in November–December 2017; this was correlated with a decrease in the number of acutely diseased animals. Concomitant to the tests aimed at the detection of BTV, we also monitored for presence of non-BTV pathogens by qRT-PCR. In contrast to findings of exams carried out in January–February 2017, where twenty-four whole blood samples from ill cattle were positive for the epizootic hemorrhagic disease virus (EHDV), no such signs of EHDV were observed in August–October 2017. Regarding BEFV, a mini outbreak was registered in one geographic locality situated in the coastal area of Israel, where BEFV was detected in seven milking cows in late July–early August 2017. All samples from cattle, where BTV-6 was identified, bar one which was not tested for BEFV, were negative for BEFV by qRT-PCR. Together, these data allow us to assume that neither EHDV nor BEFV were involved in the identified cases of cattle illness during summer–autumn 2017, at least in the cattle farms where BTV-6 was identified.

### 3.3. Phylogenetic, BLAST and Pairwise Analysis of BTV-6 Isolates

**Figure 2:**
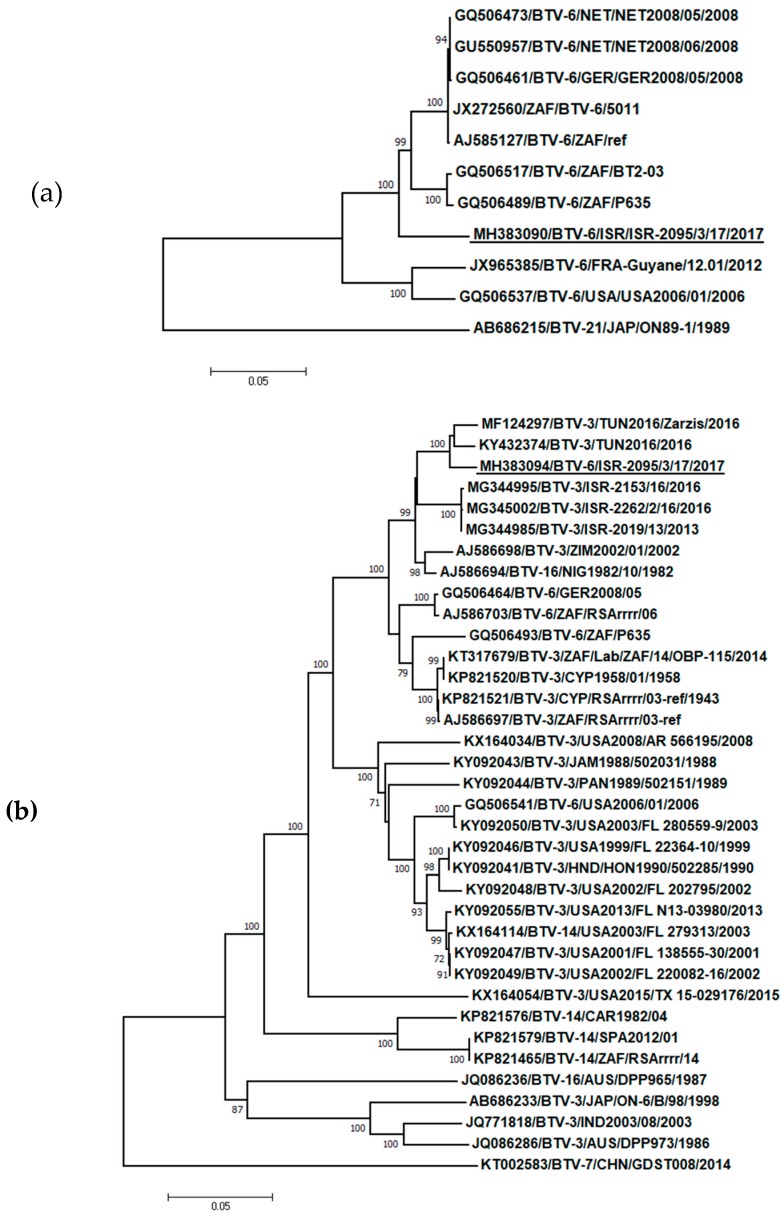
Phylogenetic analysis of Israeli BTV-6 strain with global BTV strains. (**a**) Segment 2 (VP2); (**b**) Segment 6 (VP5). The recent Israeli BTV-6 strain is underlined. Viruses were identified by accession number/serotype/location/isolate/year. The evolutionary history was inferred using the neighbor-joining method. The percentage of replicate trees in which the associated taxa clustered together in the bootstrap test (1000 replicates) with a percentage higher than 70 are shown next to the branches. The trees are drawn to scale, with branch lengths in the same units as those of the evolutionary distances used to infer the phylogenetic tree. The evolutionary distances were computed using the p-distance method and are in the units of the number of base differences per site. Codon positions included were 1st+2nd+3rd+Noncoding. All positions containing gaps and missing data were eliminated.

Partial sequencing of Segs-1, -2 -3, -5, -6, -7 and -10 2017 Israeli BTV-6 showed almost identical nucleotide sequences with the representative strain ISR-2095/3/17. Phylogenetic analysis of Seg-2 (Figure 2a), which codes for the serotype-defining protein VP2, showed that Israeli BTV-6 formed a separate branch, which clustered with BTV-6 strains originated in Africa and Europe. Specifically, the highest degree of identity (93.88%) was with South African BTV-6 reference strain 5011. Similar analysis of Seg-6, which codes for the additional outer capsid protein VP5, revealed the clustering of the Israeli BTV-6 strain with BTV-3 strains identified in Africa and Mediterranean basin (strains TUN2016, TUN2016/Zarzis, ZIM2002/01, ISR-2019/13, ISR-2153/16 and ISR-2262/2/16 in addition to the BTV-16 strain originating from Nigeria (NIG1982/10)). The notion of the genetic proximity of the Israeli BTV-6 strain with BTV-3 strains originating from Africa and the Mediterranean Basin was further supported by BLAST-based phylogenetic analysis of the sequences of Segs-1, -4, -5, and -9 (Figures S1, S3, S4 and S7). Specifically, the sequences of the Israeli BTV-6 strain showed highest degree of nucleotide sequence identity with: BTV-3/TUN2016 (Seg-1 and Seg-4, 99.47% and 99.3% identity, respectively, Table 6), BTV-3/ITL/SAR2018 (Seg-5 and Seg-9, 99.41 % and 99.13% identity, respectively, Figures S4 and S7, Table 6) and an Israeli BTV-3 isolate BTV-3/ISR-2019/13 (Seg-7, 97.78%, Table 6). Notably, Segs-3, -8 and -10 showed the highest degrees of sequence identity with other BTV serotypes, all of which originated from Africa: BTV-4/SUD1983 (Seg-3, 97.0%, Figure S2, Table 6), BTV-9/LIB2008 (Seg-8, 97.81%, Figure S6, Table 6) and a BTV isolated in Alpaca ZAF/BT 57/08 (Seg-10, 97.2%, Figure S8, Table 6). Together, these analyses support the notions of the reassortant nature of the Israeli BTV-6, and of the contiguous/proximal geographical origins (Africa and Mediterranean Basin) of the probable parental strains.

## 4. Discussion

Israel has a long history of presence and effect of BTV on livestock, beginning with the first BT clinical detections in the late 1940s [20]. In particular, BTV-6 was identified in Israel between 1972 and 1989 [20]. Unfortunately, prior to the present study, there was a lack of information about the clinical manifestation of the disease in local Israeli sheep caused by BTV-6. Such a lack of annotated information prevents a systematic comparison of our current findings on the clinical features of BTV-6 in Israeli livestock, with previous documented cases of BTV-6 in Israel. However, a comparison of our data with those obtained from BTV-6 outbreaks in Europe in 2008 reveals considerable differences. There, clinical signs of BT in cows in Netherlands were limited (e.g., to coronitis), a fact which correlated with PCR-based measurements suggestive of very low spread or a very short viremia. This is in sharp contrast to the recent Israeli BTV-6 outbreak, which quickly spread through Israel causing heavy BT clinical manifestations both in sheep and in cattle. We propose that the basis of such a difference stems from the origin and genetic composition of these different viruses, where the European outbreak was most probably derived from a live attenuated South African vaccine strain, which is considered non-virulent, while the Israeli outbreak was caused by the new BTV-6 strain that we describe here [15,16]. A comparison of clinical signs caused BTV-6 with other BTV serotypes circulated in Israeli livestock in 2017 revealed that the most prominent clinical signs both in cattle and in sheep were hyperemia and edema, mostly seen in young animals. BTV-2 and BTV-4 usually cause clinical signs in Israeli cattle (adult milk producing cows) and in the susceptible population of sheep. This is in contrast to BTV-6, where clinical signs in cattle were mostly observed in young animals. According to our analysis, a proposed major genetic source of the Israeli BTV-6 is BTV-3. Within this context, multiple recent studies have identified BTV-3 strains in the Mediterranean Basin [14,33,34,35]. Notably, the reported clinical BT manifestations of BTV-3 were in sheep only, a markedly different pattern than what was observed for the Israeli BTV-6, as our data support the notion that it affects both sheep and cattle. While serving as a basis for detection and characterization of a new BTV-6 strain in Israel, the present study is most certainly an underestimation of the full extent of Israeli distribution of BT in general, and BTV-6 in particular. This is due to the fact that it is based on passive investigation, using only diagnostic samples collected by veterinary doctors from ill animals (usually a small number of samples from affected animals in each farm, Table 4). Furthermore, the attribution of the clinical phenomena to BTV-6 is also limited by the possibility that the clinical state of the animals may have also reflected other viral and bacterial infections, which could cause pneumonia in fattening calves. Moreover, mixed BTV-3 and -6 infection were registered in at least two out of six BT-affected sheep flocks, raising the possibility that BTV-3 may have also contributed to the observed clinical symptoms.

Genetic analysis of single nucleotide mutation rates and the rate of reassortment (i.e., genetic drift and genetic shift), suggests that the latter is likely to be a major driver of genotypic and phenotypic change in BTV [28]. In this context, the unique features of Israeli BTV-6, such as the magnitude of its spread and/or its ability to induce clinical signs in both sheep and cattle, may reflect its reassortant origin, where a backbone of segments from currently circulating Mediterranean BTV-3 (Seg-1, 4, 5, 6, 7 and -9) was supplemented by contributions from BTV-6 (Seg-2), BTV-4 (Seg-3), BTV-9 (Seg-8) and an untyped BTV isolate (Seg-10). Notably, Seg-2 and Seg-10, which encode for VP2 and NS3 and have been proposed as determinants of virulence [25], were not of BTV-3 origin. Concerning the geographical origin of the proposed contributing BTV strains, we propose that co-circulation in Africa played a predominant role in generating Israeli BTV-6. This proposal also includes the segments presenting highest similitude with Italian BTV-3 isolates, as these have also been proposed to be of North African origin [33]. The notion that BTV distribution in Israel is heavily influenced by strains that circulate in North Africa is further supported by our unpublished results, which show that BTV-3 strains identified in Israel between 2013-2016 (ISR-2019/13, ISR-2153/16 and ISR-2262/2/16) also exhibit high percentages of genetic identity by some of genes with strains originating from Tunisia (Figure 2b, and Figures S2, S3 and S5). In summary, following the observation in the field of farm animals presenting BT-like symptoms, we have isolated and genetically characterized a new reassortant BTV-6 strain. This strain exemplifies how novel disease-causing abilities may stem from co-circulation and reassortment of BTV strains.

## Figures and Tables

**Table 1:** Routine molecular diagnostic investigation of field Israeli samples collected from ill and dead animals in 2017 for viral RNA presence of Bovine ephemeral fever virus (BEFV) and hemorrhagic disease virus (EHDV).

	Type of Animals					
RT-qPCR	Cattle			Wild/Zoo Animals		Positive/Total Num of Samples
	w. blood	spleen	a. fetus	spleen	a. fetus	
BEFV	7/212	0/1	0/1	NT	NT	7/214
EHDV	24/217	0/6	0/4	0/8	0/1	24/236

w. blood—whole blood EDTA samples; a. fetus—aborted fetus; Num—mumber; Positive/Total Num of samples—positive in RT-qPCR samples from total number of tested samples.

**Table 2:** Field samples, tested by Pan-BTV RT-qPCR, from different kinds of domestic and wild/zoo ill or dead animals and subsequent virus isolations during 2017.

	Cattle			Sheep			Goat				Wild Animals		
	w. b.	s.	a. f.	w. b.	s.	a. f.	w. b.	s.	w. b.	s.	a. f.	Total	total VI
													
Num of pos. samples	93	6	0	51	9	0	0	6	1	2	0	166	
Num of tested samples	402	35	7	173	41	10	9	24	17	23	3	693	
Num isolated BTV-2	1												1
Num isolated BTV-3				4									4
Num isolated BTV-4	1			12									13
Num isolated BTV-6	6			10									16
Num isolated BTV-15	6												6
Total Num VI	14			26									40

w. b.—whole blood EDTA samples; s.—spleen; a. f.—aborted fetus; num- number; VI—virus isolation; Total- total number of tested samples or virus isolates. The data are shown in the next sequence: The first row—total number of positive samples; the second row—number of tested samples; lower rows—BTV serotype/number BTV isolated in ECE.

**Table 3:** Localities and epidemiological aspects of sheep and cattle farms, where BTV-6 was identified.

Locality/Geographic Zone/Distinct	Farm	Species/ Num Animals in the Farm	Breed	Affected Group	Num of Dead Animals/ Num of Ill Animals/ Total Num of Animals in Affected Group	Morbidity/Case Mortality (%)
Yonatan/Golan Height/Northern distinct	1	sheep/500	Marino	recently bought 17-20 month-old pregnant ewes	27/78/ no data	no data/34.6
Sde David/Negev desert/Sothern distinct	2	sheep/450	Asaf x Merino	lambs of different ages	10/50/150	33.3/20
Rosh HaNikra/Golan Height/Northern distinct	3	cattle/no data	Holstein-Friesian	no data	0/no data/no data	no data
Moshav Nahalal/Jezreel Valley/Northern Distinct	4	cattle/no data	mixed breed	male fattening calves 3–7 month old	2/no data	no data/no data
Moshav Lachish/Negev Desert/Southern Distinct	5	sheep/1350	Merino× Romanov × Asaf × Puld-Dorset	lambs 4–15 month and primipara ewes	20–30/100/ no data	no data/20-30
Mishav Nehalim/Central distinct	6	sheep/no data	Marino x Safolk	6 month old	10/12/70	17.1/83.3
Kefar Blum/Galilee/Northern distinct	7	cattle/350	Holstein-Friesian	heifers	0/ no data/ 70	no data/0
Kibutz Gonen/Galilee/Northern distinct	8	cattle/350	Holstein-Friesian	heifers	0/no data/70	no data/0
Havat Shaharim/Samaria/Central distinct	9	sheep/100	mixed breed	lambs 4–6 month old	2/no data	no data/no data
Moshav Moledet/Galilee/Northern distinct	10	sheep/3500	Asaf	lambs 4–6 month old and adult animals	24/ 1200/3500/	34.3/2
Kibutz Ma’ale HaHamesha/Judean hills/Central distinct	11	cattle/500	Holstein-Friesian	5 month old female calf	0/1/50	2/0

Num—number.

**Table 4:** Clinical signs and virus isolation in BTV-6 affected cattle and sheep farms.

Farm	Date of Beginning BT Disease in the Farm	Clinical Signs/Duration	Sample Date	RT-qPCR Positive/Total	VI	Serotype
1	no data	Lameness, rejection of moving, bloody nasal discharge/no data	29-Aug-2017	3/3	2	BTV-6
2	beg-Sep-2017	Pyrexia and skin hyperemia/no data	6-Sep-2017	3/3	2	BTV-6 BTV-3
3	no data	No data/no data	12-Sep-2017	3/3	1	BTV-6
4	end-Aug-2017	Inappetence, cachexia, dyspnea and cough/two months	12-Sep-2017	2/2	2	BTV-6
5	mid-Sep-2017	Pyrexia, followed by lameness and stiffness in legs and back muscles, conjunctivitis, nasal discharge, ulceration of oral and nasal mucosa, recumbency, fatigue, mild respiratory distress and a few abortions	14-Sep-2017;28-Nov-2017	3/3; 2/2	2; 1	BTV-6 BTV-3 BTV-3
6	no data	No data/no data	19-Sep-2017	2/2	2	BTV-6
7	no data	Symmetrical bilateral edema for all length of the hind limbs; skin of udders in some infected animals was hyperemic, dry and scaly/no data	24-Sep-2017	1/1	1	BTV-6
8	no data	Same as in farm 7; cow fever and icterus in some/no data	28-Sep-2017	2/2	1	BTV-6
9	end-Sep-2017	Heavy hyperemia of udder and face/3 weeks	6-Sep-2017	1/1	1	BTV-6
10	beg-Sep-2017	Pyrexia and skin hyperemia/light perinasal and perioral edema, milk reduction/several days	3-Oct-2017	3/3	3	BTV-6
11	mid-Oct-2017	Symmetrical bilateral edema for all length of the hind limbs/two weeks	18-Oct-2017	1/1	1	BTV-6

RT-qPCR Positive/Total—positive in Pan-BTV qRT-PCR samples in total number of sent and tested samples from affected animals in the farm. VI—number of virus isolates.

**Table 5:** Ct value of positive field samples tested by pan-BTV RT-qPCR for each month during 2017 and consequent BTV isolation.

	Num of RT-qPCR positive /VI				RT-qPCR Positive/Total	BTV Serotype (VI)
Ct Value	<25.0	25.1–30.0	30.1–35.0	>35.1		
Month						
Jan	0	6/0	10/0	3/0	19/89	-
Feb	0	0	4/0	3/0	7/47	-
Mar	0	2/0	0	1/0	3/49	-
Apr	0	0	3/0	1/0	4/42	-
May	2/0	0	3/0	2/0	7/72	-
Jun	0	0	0	1/0	1/44	-
Jul	4/2	2/1	0	0	6/48	BTV-4
Aug	5/3	4/0	1/0	3/0	13/45	BTV-2, -6
Sep	16/10	12/9	10/2	4/0	42/83	BTV-3, -4, -6
Oct	5/4	11/4	7/0	1/0	24/60	BTV-3, -6, -15
Nov	2/1	3/0	15/1	7/1	27/73	BTV-3, -4, -15
Dec	0	2/0	9/2	8/0	19/41	BTV-15

Num—number; VI—virus solation; RT-qPCR Positive/Total—number of positive samples among all tested field samples during the month.

**Table 6:** Blast analysis of Israeli BTV-6 showing closest nucleotide and segment sequence identity with publically available BTV sequences.

Genome Segment/acc. Num	Closest Sequence-nt Identity (%)
Seg-1/MH383089	KY432369/BTV-3/TUN2016/2016-99.47
Seg-2/MH383090	JX272560/BTV-6/ZAF/5011-93.88
Seg-3/MH383091	DQ186825/BTV-4/SUD1983/01/1983-97.00
Seg-4/MH383092	KY432372/BTV-3/TUN2016/2016-99.33
Seg-5/MH383093	MK348541/BTV-3/ITL/SAR2018/2018-99.41
Seg-6/MH383094	MF124297/BTV-3/TUN2016/Zarzis/2016-97.62
Seg-7/MH383095	MG344986/BTV-3/ISR-2019/13/2013-97.78
Seg-8/MH383096	KP821811/BTV-9/LIB2008/03/2008-97.81
Seg-9/MH383097	MK348545/BTV-3/ITL/SAR2018/2018-99.13
Seg-10/MH383098	KP196612/ZAF/BT 57/08/2008- 97.12

Viruses were identified by accession number/serotype/location/isolate/year.

## Supplementary Materials

It can be found at https://www.mdpi.com/1999-4915/11/7/633/s1.
