# Supplementary material for: Emergence of a Novel Reassortant Strain of Bluetongue Serotype 6 in Israel, 2017: Clinical Manifestations of the Disease and Molecular Characterization
Source: Viruses. 2019 Jul 10;11(7):633. doi: 10.3390/v11070633 (PMC6669665; doi:10.3390/v11070633)
Supplement: Supplementary file 1 [file viruses-11-00633-s001.pdf]

Supplementary Materials:

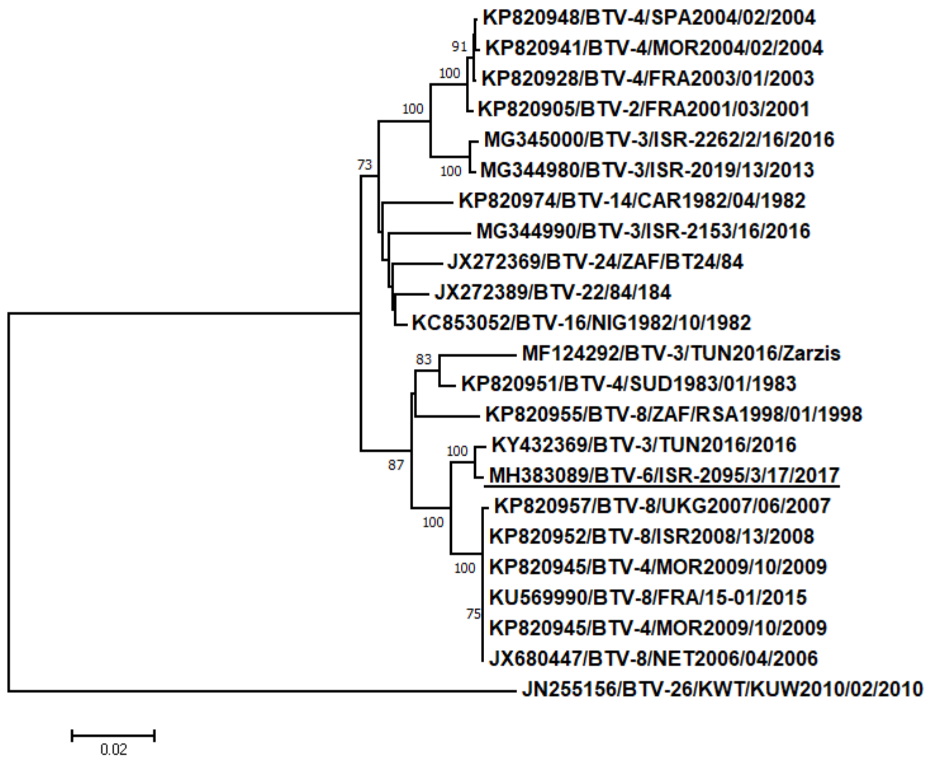

Figure S1. Phylogenetic tree of segment 1 of recently identified Israeli BTV-6 with publically available BTV strains.

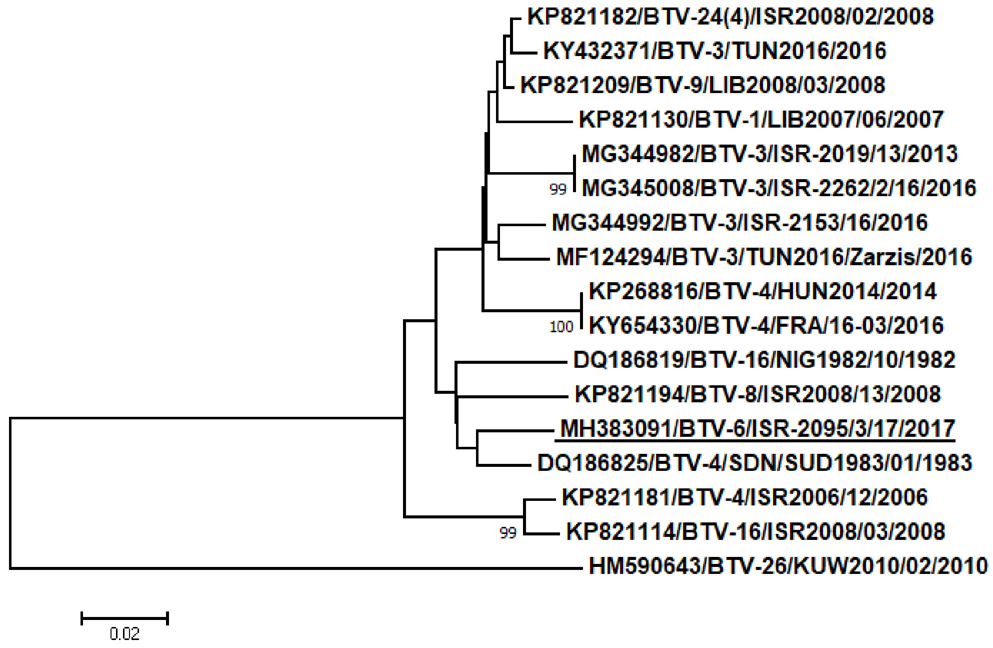

Figure S2. Phylogenetic tree of segment 3 of Israeli BTV-6 with publically available BTV strains.

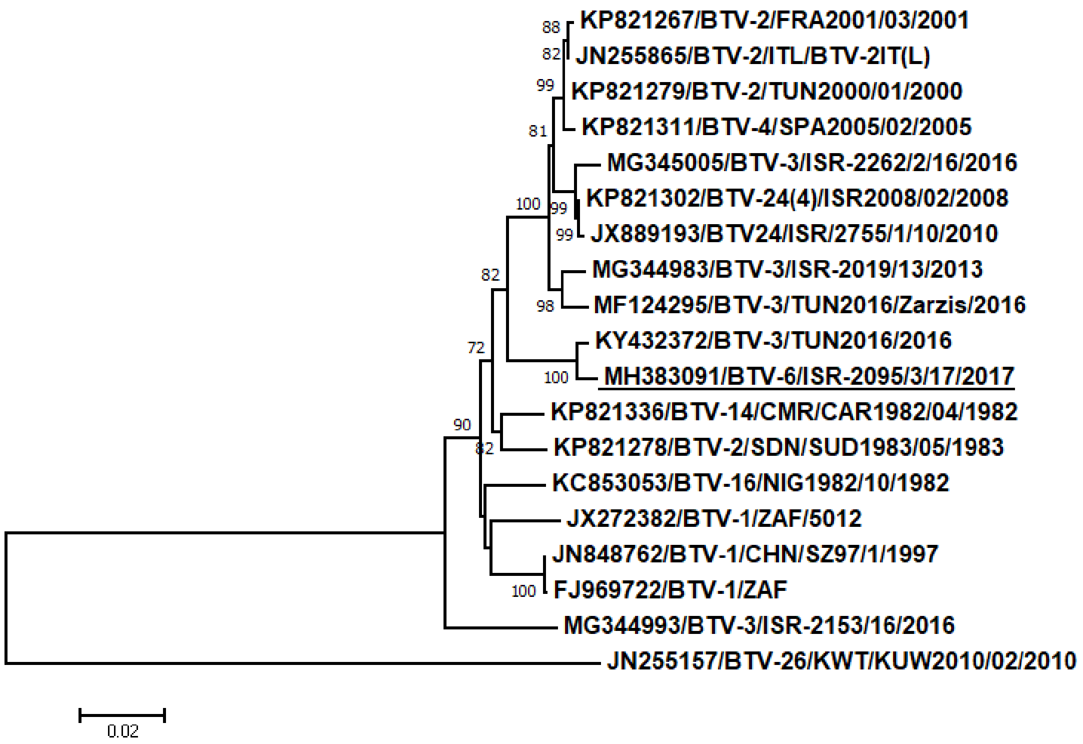

Figure S3. Phylogenetic tree of segment 4 of Israeli BTV-6 with publically available BTV strains.

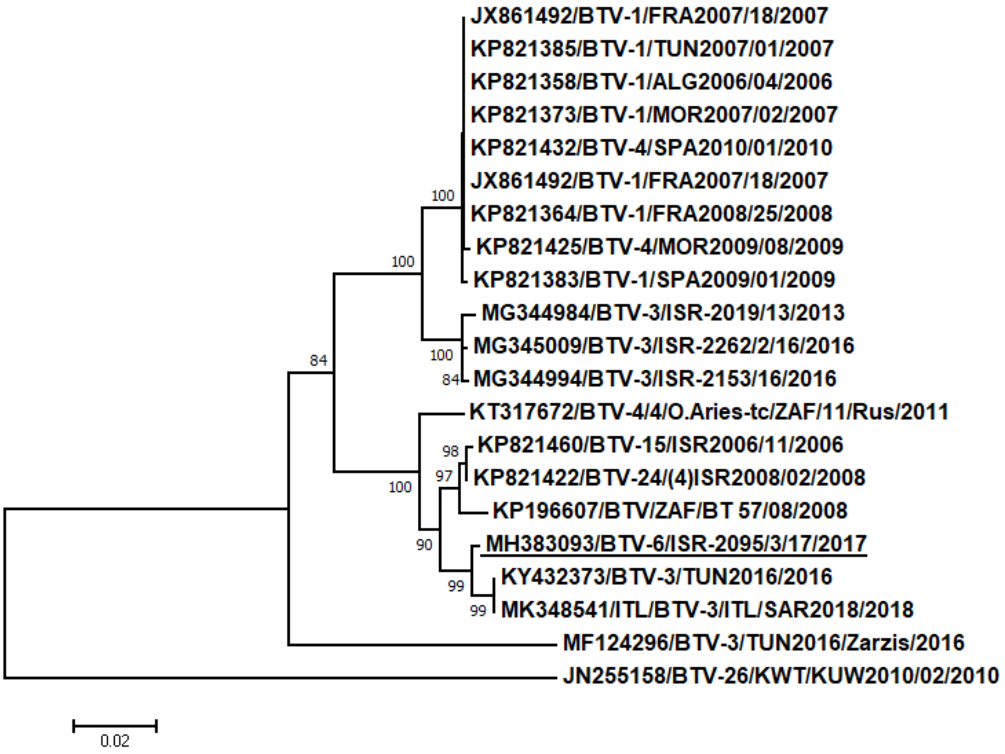

Figure S4. Phylogenetic tree of segment 5 of Israeli BTV-6 with publically available BTV strains.

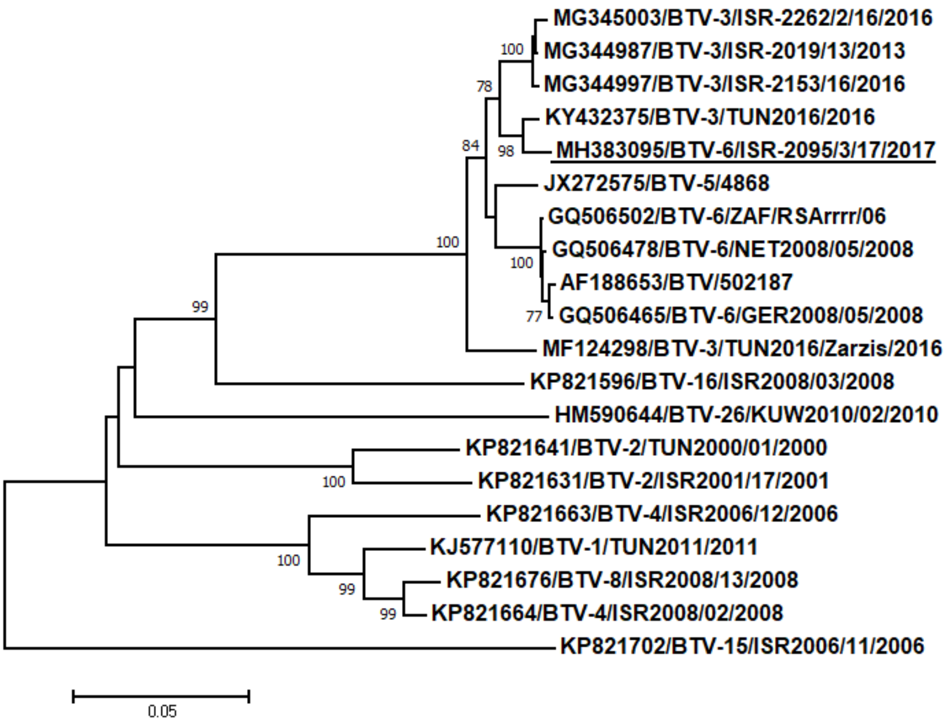

Figure S5. Phylogenetic tree of segment 7 of Israeli BTV-6 with publically available BTV strains.

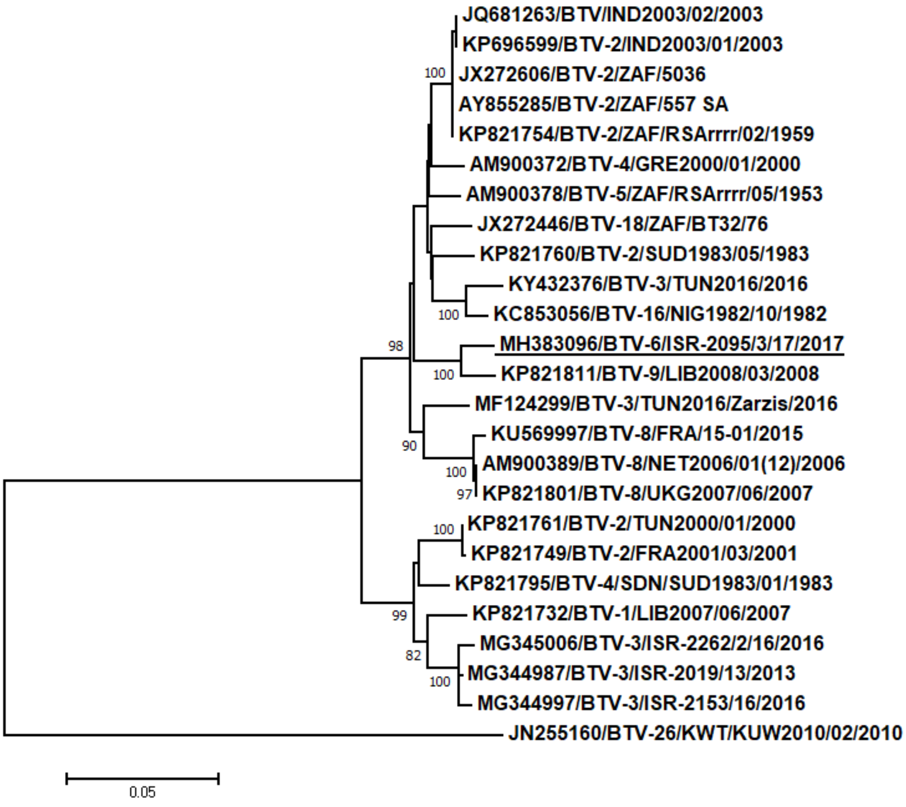

Figure S6. Phylogenetic tree of segment 8 of Israeli BTV-6 with publically available BTV strains.

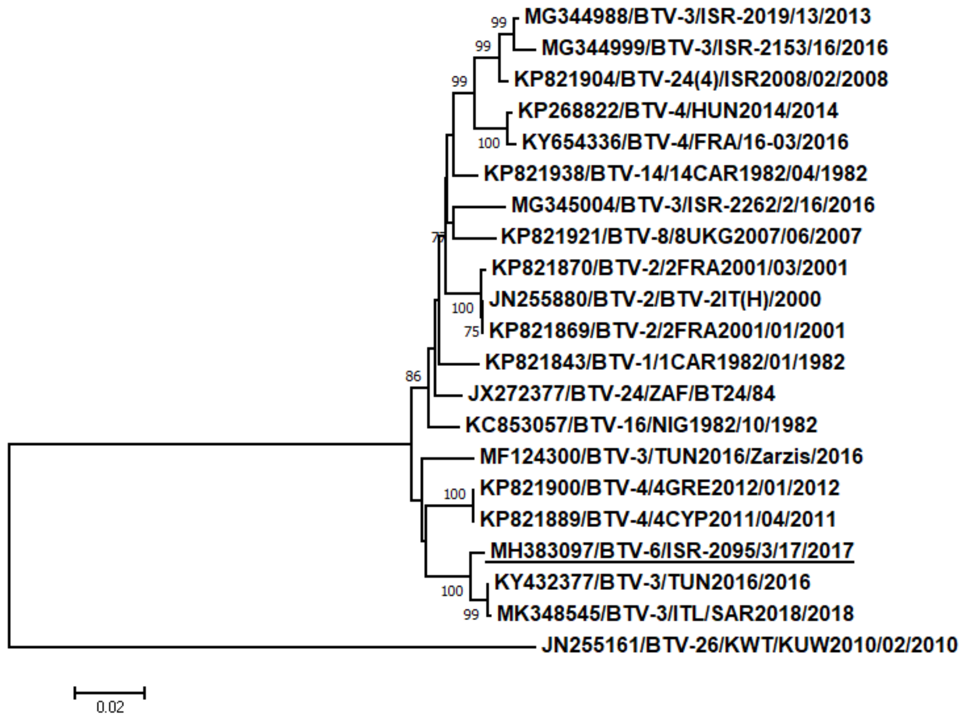

Figure S7. Phylogenetic tree of segment 9 of Israeli BTV-6 with publically available BTV strains.

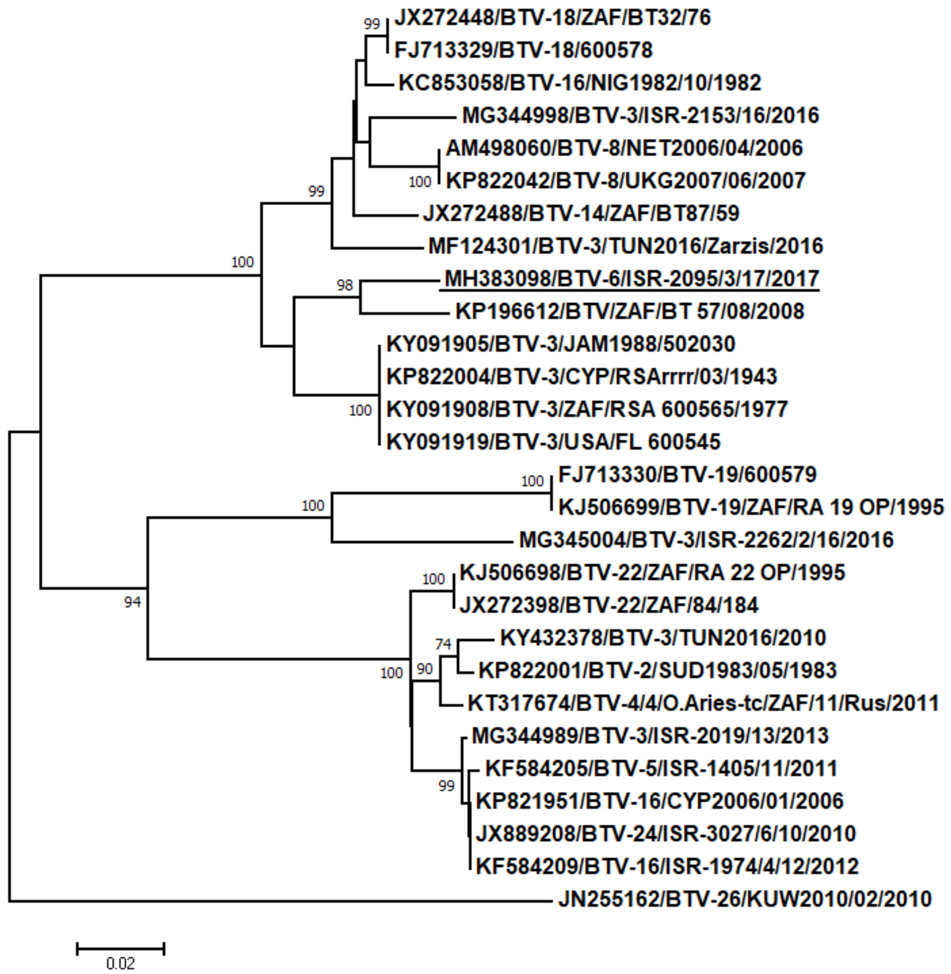

Figure S8. Phylogenetic tree of segment 10 of Israeli BTV-6 with publically available BTV strains.

Figure S1-S8: Phylogenetic analysis of Israeli BTV-6 strain with global BTV strains. Recent Israeli BTV-6 strain was underlined. Viruses were identified by accession number/serotype/ location /isolate /year. The evolutionary history was inferred using the Neighbor-Joining method. The percentage of replicate trees in which the associated taxa clustered together in the bootstrap test (1000 replicates) percentage higher than 70 were shown next to the branches. The trees were drawn to scale, with branch lengths in the same units as those of the evolutionary distances used to infer the phylogenetic tree. The evolutionary distances were computed using the p-distance method and are in the units of the number of base differences per site. Codon positions included were 1st+2nd+3rd+Noncoding. All positions containing gaps and missing data were eliminated. BTV-26 KUW2010/01 strain was used as an outgroup strain in Seg-3, 4, 5, 8, 9, and 10, when in Seg-7 BTV-15 ISR2006/11starain was used as an outgroup strain.
